# The Prophylactic Effect of Single vs. Dual Antibiotic-Loaded Bone Cement against Periprosthetic Joint Infection Following Hip Arthroplasty for Femoral Neck Fracture: An Analysis of the German Arthroplasty Registry

**DOI:** 10.3390/antibiotics12040732

**Published:** 2023-04-08

**Authors:** Dominik Szymski, Nike Walter, Paula Krull, Oliver Melsheimer, Siegmund Lang, Alexander Grimberg, Volker Alt, Arnd Steinbrück, Markus Rupp

**Affiliations:** 1 Department for Trauma Surgery, University Hospital Regensburg, 93053 Regensburg, Germany; dominik.szymski@ukr.de (D.S.);; 2 Deutsches Endoprothesenregister (EPRD) gGmbH, 10623 Berlin, Germany; 3 Orthopädisch Chirurgisches Kompetenzzentrum Augsburg (OCKA), 86152 Augsburg, Germany

**Keywords:** hip arthroplasty, hemiarthroplasty, PMMA, bone cement, periprosthetic joint infection, revision, femoral neck fracture, antibiotic-loaded bone cement, antibiotics

## Abstract

Background: Antibiotic-loaded bone cement in arthroplasties is currently experiencing increased usage. Therefore, single and double antibiotic-loaded bone cements are commercially available and used in orthopedic surgery. The aim of this investigation was to compare the clinical use of single compared to dual antibiotic-loaded bone cement for implant fixation after femoral neck fracture. Further infection rates were to be compared in (partial) arthroplasty for the treatment of femoral neck fracture for both treatment options. Methods: On the basis of the German Arthroplasty Registry (EPRD), all cases of femoral neck fracture treated with hemiarthroplasty (HA), or total hip arthroplasty (THA) with single and dual antibiotic-loaded bone cement, were included into the data analysis. The infection risk was compared using Kaplan-Meier estimates. Results: In total, 26,845 cases (HA 76.3%–THA: 23.7%) with femoral neck fracture were included. Within recent years, an increasing usage of dual antibiotic-loaded cement in Germany, with a current proportion of 7.30% in arthroplasty procedures for femoral neck fracture treatment, has been observed. In patients treated with HA, the proportion of dual antibiotic-loaded cement was 7.86%, while in those treated with THA, 5.46% of all prostheses were fixated with a two antibiotic component cement. For all arthroplasty procedures using single antibiotic-loaded bone cement after six months 1.8%, after one year 1.9%, and after five years 2.3%, of the cases failed due to periprosthetic joint infection (PJI), while in the same time period, in cases with dual antibiotic-loaded bone cement 1.5%, 1.5% and 1.5% suffered from infection (*p* = 0.34). A infection rate of 1.1% after HA with dual antibiotic-loaded bone cement was reported, compared to a 2.1% infection rate whilst using single antibiotic-loaded bone cement after five years (*p* = 0.098). The number required for treatment when using HA was 91. Conclusions: The use of dual antibiotic-loaded bone cement is increasingly used in arthroplasty procedures after femoral neck fractures. It demonstrates a reduction of PJI after HA and seems, therefore, to be a useful method for the prevention of infection, especially in patients with increased risk factors for PJI.

## 1. Introduction

Projections of intracapsular femoral neck fractures estimate up to 21.3 million fractures will occur annually, with a doubling of these totals occurring worldwide between 2025 and 2050 [1]. In the U.S. only, approximately 800,000 femoral neck fractures occur yearly and cause direct healthcare costs between $14,776 and $17,097, placing a tremendous burden on the healthcare system [2,3]. In Germany, the same issue was reported. A significant increase of femoral neck fracture cases, of 23.3% within one decade, indicated that it is the most frequently surgically treated fracture [4]. Femoral neck fractures and mostly treated by partial (hemiarthroplasty (HA)) or total hip arthroplasty (THA). With regards to the type of fixation for hemiprostheses, geographical differences exist. In Europe, mainly cemented Has are implanted (up to 90%), whereas, in the U.S., cementless HA fixation is preferred [4,5]. A similar trend was observed in the use of antibiotic-loaded bone cement. While in Europe, almost exclusively, preparation is completed with the addition of one or two antibiotics (mainly gentamicin or gentamicin in combination with clindamycin or vancomycin), in the U.S., the application of antibiotics in bone cement was only reported in 44% of the cases according to the American Joint Replacement Registry [5,6]. 

One major complication of arthroplasty is the development of a periprosthetic joint infection (PJI), which occurs in up to 3% of the performed (partial) arthroplasties [7,8]. Besides a high psychological burden for patients and their families, PJI often need a two-stage revision procedure and therefore demonstrate immense socioeconomic costs for the healthcare systems [9,10]. The use of antibiotic-loaded bone cement has been reported to lead to a significant reduction in the rate of infections by up to 72.6%, with cost savings of up to $3500 [11,12]. Sprowson et al. (2016) investigated, in a quasi-randomized study of 848 patients with femoral neck fracture, the effect of dual antibiotic-loaded bone cement compared to single antibiotic-loaded bone cement, and registered a reduction of infection rate by 2.4% (*p* = 0.041) within one year after HA implantation [13]. However, there is a lack of long-term data representing a large patient collective analyzing the use and effect of dual antibiotic-loaded bone cement compared to single antibiotic-loaded bone cement on the development of PJI in a population of patients treated with (partial) arthroplasty after intracapsular femoral neck fracture. 

Therefore, the aim of the present investigation was (1) the detection of trends in used bone cements with antibiotics in a single or dual-loaded manner for HAs and THAs for the treatment of intracapsular femoral neck fractures since 2012. Additionally, (2) infection rates after cemented HAs and cemented THAs for intracapsular femoral neck fracture using single and dual-antibiotic loaded bone cement should be elucidated. 

## 2. Results

In this investigation, overall 26,845 cases of arthroplasty treatment were included. Thereby, 20,487 (76.3%) of cemented HAs and 6358 (23.7%) THAs after intracapsular femoral neck fracture were analyzed. Anthropometric data was summarized in Table 1. 

**Figure 1:**
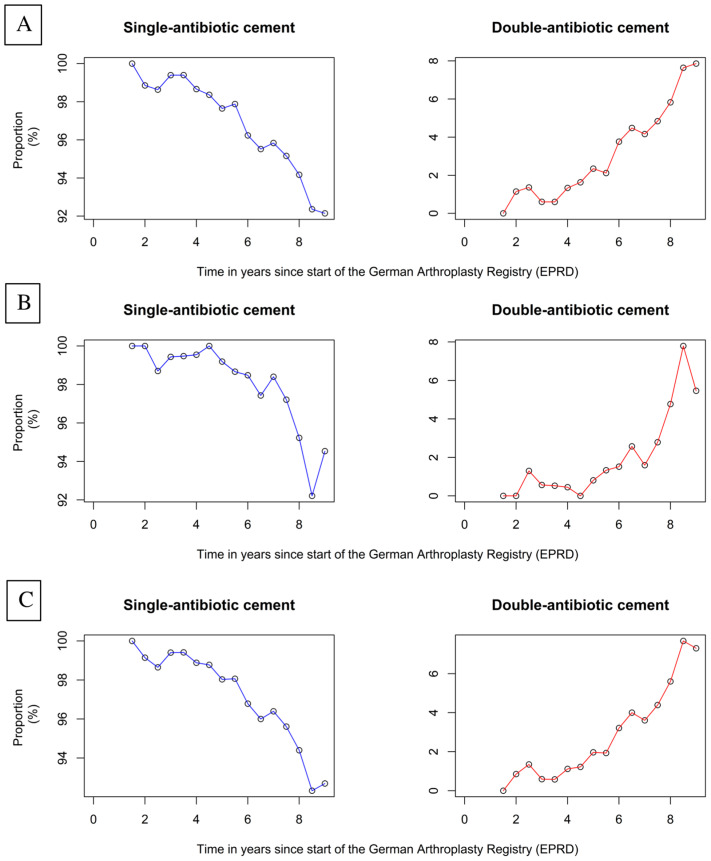
Usage of single and dual antibiotic-loaded cement after arthroplasty treatment for femoral neck fracture (HA + THA) (**A**), HA only (**B**) and THA only (**C**) since the start of the German Arthroplasty Registry (EPRD).

Within nine years since the start of the German Arthroplasty Register (EPRD) in 2012, for HAs and THAs a clear trend of increased usage of dual antibiotic-loaded bone cement has been reported. In HAs, the proportion of dual antibiotic-loaded bone cement raised from 1.15% in the second year of the register to 7.86% after nine years of the register. In cemented THAs the rate of dual antibiotic-loaded bone cement raised from 1.30% to 5.46% (Figure 1).

**Figure 2:**
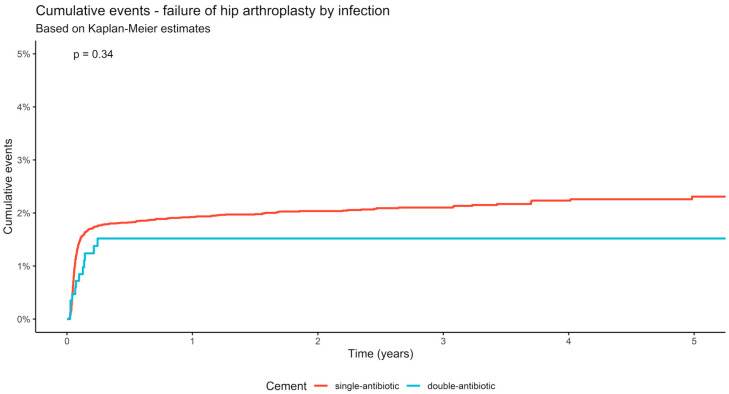
Infection rate after single and dual antibiotic-loaded bone cement cumulative in hemiarthroplasty and total hip arthroplasty after femoral neck fracture.

For all cases of intracapsular femoral neck fracture, with treatment by cemented HA or THA, a reduction in the number of infections was observed, comparing the use of dual antibiotic-loaded bone cement with single antibiotic-loaded bone-cement (*p* = 0.34). In single antibiotic-loaded bone cement the cumulative infection incidence was 1.3 % after one month, 1.8% after six months, and 2.3% after five years. In the same time period for arthroplasties with dual antibiotic-loaded cement, infection rates of 0.7%, 1.5%, and 1.5% were reported. The number needed for the prevention of just one infection was 125 after 5 years (Table 2; Figure 2). 

**Figure 3:**
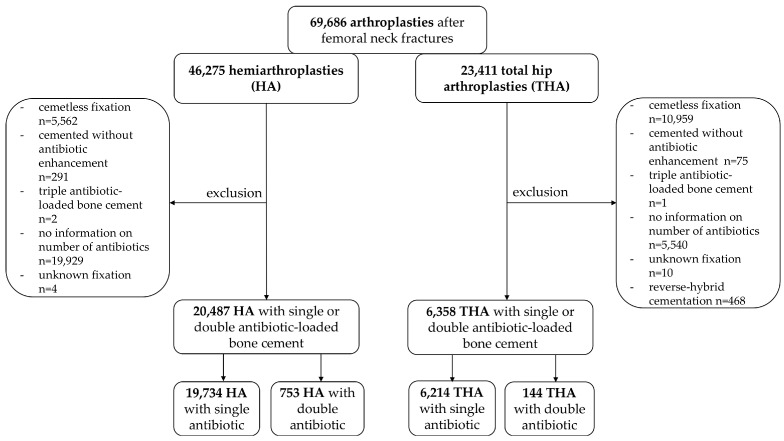
Flow chart of patient inclusion and exclusion criteria.

In patients treated with cemented HA after intracapsular femoral neck fracture, no statistically significant difference between single antibiotic-loaded and dual antibiotic-loaded bone cement was reported (*p* = 0.098). The proportion of infection raised from 1.8% in single antibiotic-loaded cement, and 1.1% in dual antibiotic-loaded cement after six months, to 1.9% and 1.1%, respectively, after one year, and 2.1% and 1.1%, respectively, after five years (Figure 3).

Patients treated with a cemented THA also demonstrated no significant differences between single antibiotic-loaded bone cement and dual antibiotic-loaded bone cement (*p* = 0.17). After six months, in the subpopulation of single antibiotic-loaded bone cement, a proportion of 1.8% infections was reported, while in dual antibiotic-loaded bone cement a rate of 3.8% of infections were documented. One year after implantation, 1.9% were registered in single antibiotic-loaded, and 3.8% in the dual antibiotic-loaded bone cement, whereas after five years 2.7% and 3.8% were reported, respectively. The number needed for the prevention of just one infection was 91 in HA after 5 years (Table 3). 

## 3. Discussion

In this retrospective analysis of 26,845 intracapsular femoral neck fractures treated with (partial) arthroplasty registered in the German Arthroplasty Register (EPRD), a trend with an increase of 7.3% within the last nine years was observed for the use of dual antibiotic-loaded bone cement. While no statistically significant differences with regards to rate of infection were reported for the usage of single antibiotic-loaded and dual antibiotic-loaded bone cement, the proportion of infection was lower for the treatment with dual antibiotic cement. The number needed to treat in order to prevent one PJI, using dual antibiotic-loaded bone cement for HA, was 91.

Since the start of the German Arthroplasty Register a clear increase in the use of dual antibiotic-loaded bone cement was detected within the period of 2013 until 2022. For HA and THA, over 7% of prostheses were implanted using dual antibiotic-loaded bone cement. The rate of dual antibiotic-loaded bone cement was even higher in HA, with a figure of 7.86%. Recent published studies reporting an improved effect on surgical site infection prevention and increased marketing activities of companies might be the reason for this observed trend in recent years [13,14,15,16]. Furthermore, improved release kinetics of antibiotics using dual antibiotic-loaded bone cement, as found in the combination of gentamicin with clindamycin, might be a scientific explanation for this clinical trend being observed [17]. 

A reduction in the rate of PJI was detected across the five-year study period for the use of dual antibiotic-loaded bone cement compared to the single antibiotic-loaded bone cement for HA for the treatment of intracapsular femoral neck fractures. After five years, the difference in infection rate in HA was 1.0%. For all arthroplasties the number needed to treat was calculated with 125 cases for the prevention of one PJI; in HA, the number needed to treat was 91. Because of the burden and long-term consequences of PJI the prevention of infection is extraordinarily relevant. Due to the small number of THA cases in which dual antibiotic-loaded bone cement was used, calculated differences, infection rates and, especially the number needed to treat in this group should be regarded with caution. Higher numbers acquired in the coming years will surely draw a clearer picture for this special patient cohort.

In HA, a greater benefit is expected for dual antibiotic-loaded cement compared to THA, due to the more vulnerable patient population. The indication for implantation of an HA occurs not only because of advanced age and having a shortened life expectancy (<5 years), but also as a result of the presence of relevant comorbidities. In particular, this patient population is at risk of developing PJI [18,19]. Previous investigations reported a reduction by 2.4% in patients with a high-dose dual antibiotic-loaded bone cement compared to a low-dose single antibiotic-loaded bone cement (3.5 vs. 1.1%) in HA one year after surgery. Within this time frame, and among the 848 included patients, the number needed to treat with dual antibiotic-loaded cement to prevent one infection was 42 [13]. The same study group extended their investigation and analyzed retrospectively 1941 patients with HA after femoral neck fracture. Here, a significant reduction of the PJI rate by 2.2% was detected for the treatment with dual antibiotic-loaded bone cement (*p* = 0.003) [14]. For the treatment of PJI, also, a reduction (−13%) in the number of further surgical site infections was reported for the use of dual antibiotic-loaded bone cement in one-stage revisions of infected total knee arthroplasties and THAs [20]. Furthermore, for aseptic revisions of knee arthroplasties, a significant reduction of the infection risk was detected (*p* = 0.035) with a mean assumption of cost savings of approximately USD 1367 for the inhouse treatment by prevention of PJI [15]. Compared to plain bone cement, antibiotic-loaded products demonstrated a significant reduction of PJI by 72.6% in HA and THA (*p* = 0.009) [11]. This prophylactic effect was also reflected in socioeconomic aspects with a cost saving of EUR 2672 per case for the prevention of PJI in hip arthroplasties [11]. 

Concerns about the use of antibiotic-loaded bone cement exist, particularly with regards to the development of antibiotic resistance and toxic properties. The application of antibiotic-loaded bone cement provides local high doses of antibiotics in the affected joint, and works complementary to the systematic antibiotic prophylaxis for the reduction of PJI and the treatment of potentially resistant bacteria, as well as the establishment of biofilms [21,22]. A common fear of orthopedic surgeons is the development of pathogens with antibiotic resistance after the use of antibiotic-loaded bone cement. Tootsi et al. (2021) focused in their retrospective multicenter study on this issue. In cases with confirmed PJI, no correlation between the application of antibiotic-loaded bone cement and the occurrence of antibiotic resistance was observed (OR:0.79; *p* = 0.469) [23]. These findings were confirmed by studies investigating the use of dual antibiotic-loaded bone cement [14]. Infections with clostridium difficile and renal failure are also potential and feared side effects, which, however, could not be confirmed in a randomized controlled investigation. Contrariwise, in the intervention group using dual antibiotic-loaded bone cement, a significant reduction in the number of critical care unit stays was found [13]. Taking the concerns of antibiotic-loaded bone cement, and the advantages, such as no increased rate of emerging antibiotic resistance, toxicological issues, and identical mechanical stability with a significant reduction of infection rate, into correlation, a clear clinical recommendation for the use of these products in order to prevent PJI has to be stated [23,24,25]. 

The use of bone cement in antibiotic application is industrially provided and FDA-approved. Single loaded cements thereby mostly consist of gentamicin (0.5 g or 1 g) or tobramycin (1 g), while for dual antibiotic-loaded bone cements, gentamycin is combined with clindamycin (1 g gentamicin + 1 g clindamycin) or with vancomycin (0.5 g gentamicin + 2 g vancomycin) [16,21]. Gentamicin covers the spectrum of gram-positive staphylococci, enterococci, and several gram-negative germs, while clindamycin seems useful as a good bone penetrating antibiotic. The combination of gentamicin and clindamycin provides an antibiotic coverage of up to 90% of the germs found in PJI [16,21,26]. The combination of vancomycin and gentamicin has been demonstrated to be even more efficient in the treatment of PJI. Other, also very efficient and useful, antibiotic combinations for dual-loaded antibiotic bone cements seem useful in the presence of emerging antibiotic resistances worldwide and should therefore be implemented in the market of available antibiotic-loaded bone cements [27]. 

The present registry data confirm the risk reduction of PJI after HA for femoral neck fracture according to the first results of clinical studies investigating this issue. The present data indicate that in patients with risk factors for the development of PJI dual antibiotic-loaded bone cements seem beneficial [11]. Therefore, the exact vulnerable patient cohorts who benefit from application of dual antibiotic-loaded bone cements should be identified. An increased body mass index, individual or in increased numbers of comorbidities should be further evaluated in this regard [28]. The efficacy demonstrated for arthroplasty after femoral neck fracture also implicates the need to investigate and carefully evaluate the use of dual antibiotic-loaded bone cements for even more frequently performed THA, due to osteoarthritis and other diagnoses requiring hip replacement, at least in vulnerable cohorts. 

Besides the multiple advantages of this register study, some limitations occur within the type of design and methods. One major limitation is the case number of dual antibiotic-loaded bone cement in THAs. These low numbers do not allow for a reliable conclusion for THA alone, in particular, with regards to the infection rate and the number needed to treat. Due to the unmatched patient collective between HA and THA, and differences between patient anthropometry, a comparison with regards to the PJI rate should only be made with caution because of the use of a different anthropometry. However, the present investigation offers some first insights into an issue with high clinical and scientific relevance, which led the authors to report the present findings. Furthermore, several questions such as the optimal patient population for dual antibiotic-loaded bone cement after arthroplasty, or the evaluation of local side effects cannot yet be answered based on the current available dataset. 

## 4. Materials and Methods

### 4.1. Data Collection 

The German Arthroplasty Registry (EPRD) registers implantations of prosthesis in collaboration with the statutory health insurance funds in Germany (AOK Bundesverband GbR, Verband der Ersatzkassen e.V vdek), the German Medical Technology Association (BVMed), and several participating hospitals since 2012. With more than 1.6 million procedures documented, the register covered, on a voluntary basis in the year 2020, approximately 70% of all hip and knee arthroplasties performed in Germany [29]. Approximately 65% of the German population are included into the data collection by the collaboration with the two participating health insurance associations (AOK-B, vdek), and the cross-validation of information by surgeons. Surgical revisions registered in the EPRD are followed up based on insurance billing data, even if performed in a hospital not participating in the arthroplasty registry. With the exception of procedures performed outside of Germany, this algorithm ensures the near-perfect tracking of patients insured by these companies [30]. 

For the classification and identification of diagnoses and procedures, the German versions of the International Classification of Procedures in Medicine (ICPM), the “Operation and Procedure Code” (OPS) 301 system, and the 10th International Classification of Diseases (ICD-10) were used. 

### 4.2. Patients

All patients with cemented HA and THA, after intracapsular femoral neck fracture as a main diagnosis (ICD-10: S72.0-), between 2013 and 2022 were included in the present analysis of the EPRD. Patients were divided into subpopulations with single antibiotic-loaded bone cement and dual antibiotic-loaded bone cement for the fixation of THA or HA. THA with hybrid cemented components were also part of the study population. All patients with a cemented fixation of components received antibiotic-loaded bone cement. Through analysis of the used bone cement, a statement regarding single or dual antibiotic-loaded bone cement was obtained (Figure 3). The infection rate was determined by the analysis of revision reasons through a search of the ICD-10 code for periprosthetic infection (T84.5) in the registry. Through this protocol, all types of septic revision surgeries were included. Exclusion criteria were patients who were not treated with a femoral neck fracture as a main diagnosis. Prostheses with hybrid-reverse cementation were also excluded from the study population. Patients in whom no statement could be made regarding the fixation of components, number of antibiotics used in bone cement, or type of prothesis were also excluded from the data collection. Prosthesis with no antibiotic-loaded bone cement and/or antibiotic-loaded bone cement with more than two enhanced antibiotics were also excluded.

### 4.3. Statistical Analysis

The data were analyzed to determine infection rates for cemented hemiarthroplasties (HA) and total hip arthroplasties (THA) after femoral neck fractures were treated with single and dual antibiotic-loaded bone cement in Germany. The statistical program R (R Foundation for Statistical Computing, Vienna, Austria) was used to perform the statistical analysis. For statistical analysis, Kaplan-Meier estimates were calculated, log-rank tests were performed, and the number needed to treat (NNT) were calculated for the data. Categorical variables are presented in the number of observations and frequency, continuous variables in mean and standard deviation. The significance level was set at alpha = 0.05.

## 5. Conclusions

The use of dual antibiotic-loaded bone cement in HA for the treatment of intracapsular femoral neck fractures demonstrated a reduction in the infection rate compared to single antibiotic-loaded cement. The observed reduction of infection rate and no evidenced side effects after the local use of antibiotic-loaded bone cement reported in the literature advocate the use of dual antibiotic-loaded bone cement, especially in patients with an increased risk of PJI after partial arthroplasty procedures for treatment of femoral neck fractures.

## Figures and Tables

**Table 1:** Anthropometry and risk factors of the patient collective with hemiarthroplasty after femoral neck fracture.

	Hemiarthroplasty (HA)		Total Hip Arthroplasty (THA)	
	Single Antibiotic-Loaded Cement	Dual Antibiotic-Loaded Cement	Single Antibiotic-Loaded Cement	Dual Antibiotic-Loaded Cement
Number (n)	19,734	753	6214	144
Age (years), Mean (SD)	84.2 (7.19)	83.1 (7.77)	78.3 (8.47)	76.9 (8.91)
Sex (female), n (%)	14,274 (72.3%)	527 (70.0%)	4580 (73.7%)	107 (74.3%)
Body-Mass-Index in kg/m^2^, Mean (SD)	24.5 (4.10)	24.4 (4.42)	24.9 (4.27)	25.0 (4.87)
Elixhauser-Comorbidity score, Mean (SD)	8.33 (7.70)	8.66 (8.13)	6.20 (7.09)	6.89 (7.93)
Revision for infection (T84.5), n (%)	349 (1.8%)	7 (0.9%)	131 (2.1%)	5 (3.5%)
Revision overall, n (%)	773 (3.9%)	18 (2.4%)	334 (5.4%)	8 (5.6%)

**Table 2:** Cumulative incidence of infection for single and dual antibiotic-loaded cement in hemiarthroplasty and total hip arthroplasty after femoral neck fracture.

	1 Month	2 Months	3 Months	6 Months	1 Year	3 Years	5 Years
**Single antibiotic-loaded bone cement** Cumulative events (%) (95% confidence interval)	1.3 (1.2–1.5)	1.7 (1.5–1.8)	1.8 (1.6–1.9)	1.8 (1.7–2.0)	1.9 (1.7–2.1)	2.1 (1.9–2.3)	2.3 (2.1–2.6)
**Dual antibiotic-loaded bone cement** Cumulative events (%) (95% confidence interval)	0.7 (0.1–1.3)	1.2 (0.5–2.0)	1.5 (0.7–2.4)	1.5 (0.7–2.4)	1.5 (0.7–2.4)	1.5 (0.7–2.4)	1.5 (0.7–2.4)

**Table 3:** Cumulative incidence of infection for single and dual antibiotic-loaded cement for HA and THA after femoral neck fracture.

		1 Month	2 Months	3 Months	6 Months	1 Year	3 Years	5 Years
**Hemiarthroplasty** Cumulative events (%) (95% confidence interval)	**Single antibiotic-loaded bone cement**	1.3 (1.1–1.5)	1.7 (1.5–1.9)	1.8 (1.6–2.0)	1.8 (1.6–2.0)	1.9 (1.7–2.1)	2.0 (1.8–2.3)	2.1 (1.9–2.3)
	**Dual antibiotic-loaded bone cement**	0.6 (0.0–1.1)	0.9 (0.2–1.6)	1.1 (0.3–1.8)	1.1 (0.3–1.8)	1.1 (0.3–1.8)	1.1 (0.3–1.8)	1.1 (0.3–1.8)
**Total hip arthroplasty** Cumulative events (%) (95% confidence interval)	**Single antibiotic-loaded bone cement**	1.4 (1.1–1.7)	1.6 (1.3–1.9)	1.8 (1.4–2.1)	1.8 (1.5–2.2)	1.9 (1.6–2.3)	2.2 (1.8–2.6)	2.7 (2.2–3.3)
	**Dual antibiotic-loaded bone cement**	1.5 (0.0–3.4)	3.0 (0.1–5.9)	3.8 (0.5–7.1)	3.8 (0.5–7.1)	3.8 (0.5–7.1)	3.8 (0.5–7.1)	3.8 (0.5–7.1)

## Data Availability

Data available on request.
